# Characterization of COVID-19 cases in the early phase (March to July 2020) of the pandemic in Kenya

**DOI:** 10.7189/jogh.12.15001

**Published:** 2022-12-30

**Authors:** Philip Ngere, Joyce Onsongo, Daniel Langat, Elizabeth Nzioka, Faith Mudachi, Samuel Kadivane, Bernard Chege, Elvis Kirui, Ian Were, Stephen Mutiso, Amos Kibisu, Josephine Ihahi, Gladys Mutethya, Trufosa Mochache, Peter Lokamar, Waqo Boru, Lyndah Makayotto, Emmanuel Okunga, Nollascus Ganda, Adam Haji, Carolyne Gathenji, Winfred Kariuki, Eric Osoro, Kadondi Kasera, Francis Kuria, Rashid Aman, Juliet Nabyonga, Patrick Amoth

**Affiliations:** 1 Department of Disease Surveillance and Epidemic Response, Ministry of Health, Kenya; 2 Washington State University, Global Health, Kenya; 3 World Health Organization, Nairobi Kenya; 4 Public Health Emergency Operation Centre, Ministry of Health, Kenya; 5 Department of Promotive and Preventive Health, Ministry of Health, Kenya; 6 National Public Health Laboratory Services, Ministry of Health, Kenya; 7 Office of the Director General, Ministry of Health, Kenya; 8 Field Epidemiology and Laboratory Training Program, Ministry of Health, Kenya; 9 Directorate of Public Health, Ministry of Health, Kenya; 10 Cabinet Administrative Secretary, Ministry of Health, Kenya

## Abstract

**Background:**

Kenya detected the first case of COVID-19 on March 13, 2020, and as of July 30, 2020, 17 975 cases with 285 deaths (case fatality rate (CFR) = 1.6%) had been reported. This study described the cases during the early phase of the pandemic to provide information for monitoring and response planning in the local context.

**Methods:**

We reviewed COVID-19 case records from isolation centres while considering national representation and the WHO sampling guideline for clinical characterization of the COVID-19 pandemic within a country. Socio-demographic, clinical, and exposure data were summarized using median and mean for continuous variables and proportions for categorical variables. We assigned exposure variables to socio-demographics, exposure, and contact data, while the clinical spectrum was assigned outcome variables and their associations were assessed.

**Results:**

A total of 2796 case records were reviewed including 2049 (73.3%) male, 852 (30.5%) aged 30-39 years, 2730 (97.6%) Kenyans, 636 (22.7%) transporters, and 743 (26.6%) residents of Nairobi City County. Up to 609 (21.8%) cases had underlying medical conditions, including hypertension (n = 285 (46.8%)), diabetes (n = 211 (34.6%)), and multiple conditions (n = 129 (21.2%)). Out of 1893 (67.7%) cases with likely sources of exposure, 601 (31.8%) were due to international travel. There were 2340 contacts listed for 577 (20.6%) cases, with 632 contacts (27.0%) being traced. The odds of developing COVID-19 symptoms were higher among case who were aged above 60 years (odds ratio (OR) = 1.99, *P* = 0.007) or had underlying conditions (OR = 2.73, *P* < 0.001) and lower among transport sector employees (OR = 0.31, *P* < 0.001). The odds of developing severe COVID-19 disease were higher among cases who had underlying medical conditions (OR = 1.56, *P* < 0.001) and lower among cases exposed through community gatherings (OR = 0.27, *P* < 0.001). The odds of survival of cases from COVID-19 disease were higher among transport sector employees (OR = 3.35, *P* = 0.004); but lower among cases who were aged ≥60 years (OR = 0.58, *P* = 0.034) and those with underlying conditions (OR = 0.58, *P* = 0.025).

**Conclusion:**

The early phase of the COVID-19 pandemic demonstrated a need to target the elderly and comorbid cases with prevention and control strategies while closely monitoring asymptomatic cases.

Coronavirus disease 2019 (COVID-19), an infectious disease caused by the severe acute respiratory syndrome coronavirus 2 (SARS-CoV-2), was first reported in December 2019 in Wuhan City, China, as clusters of pneumonia of unknown origin and spread rapidly across the globe [1]. The World Health Organization (WHO) declared COVID-19 a public health emergency of international concern (PHEIC) in January, 2020, and a pandemic in March 2020 [2]. As of July 27, 2020, a total of 16 114 449 cases and 646 641 deaths (case fatality rate (CFR) = 4.0%) globally had been reported to WHO, including 712 920 cases and 11 900 deaths (CFR = 1.7%) in Africa and 17 975 cases and 285 deaths (CFR = 1.6%) in Kenya [3,4]. This phase of the pandemic was driven by the importation of cases and sporadic isolated cluster outbreaks in Kenya. The prevention and control strategies implemented at that time were traveller screening at points of entry (POEs); movement restriction in and out of hotspots, targeted (cluster) mass testing, mandatory quarantine of travellers and all contacts, and institutional isolation of all cases [4].

The COVID-19 disease presents with a non-pathognomonic spectrum of symptoms, ranging from asymptomatic cases to mild and severe illnesses affecting several body organs and systems[5]. The incubation period is five to six days, with a range of one to 14 days; the commonly reported symptoms are fever, cough, difficulty in breathing, general malaise, sore throat, diarrhoea, loss of appetite, loss of smell (anosmia), loss of taste (ageusia), and headache [6,7]. The severe forms of the disease (including respiratory distress, respiratory failure, vital organ injuries, shock, and death) have been particularly documented among those with underlying conditions such as diabetes, chronic respiratory, cardiovascular, and gastrointestinal diseases, and hypertension; and the elderly [8-10].

As the pandemic evolved from sporadic clusters to widespread (community) transmissions, so did the need to urgently expand public health interventions to mitigate its adverse impact grow [11]. Kenya’s testing strategy focused on high-risk groups such as frontliners (including health care workers, disciplined forces, academic staff members, etc.), travellers, and close contacts of confirmed cases. This testing strategy yielded several asymptomatic cases, yet there was no data on their clinical progression, infectiousness, and duration of virus shading. In this study, we describe the characterization of the COVID-19 cases reported in Kenya in the pandemic’s early phase to understand the epidemiology and provide information necessary for monitoring the pandemic and developing prevention and control strategies.

## METHODS

**Figure 1:**
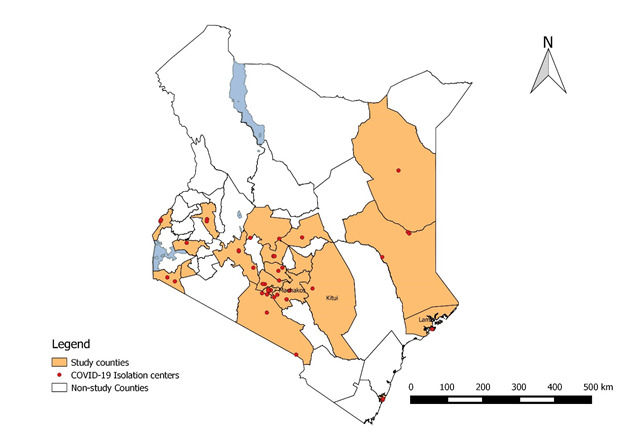
COVID-19 characterization study counties and isolation sites, Kenya, 2020.

We analysed the data of COVID-19 cases in Kenya from March through to July 2020. Data was abstracted from records of COVID-19 cases admitted in isolation centres in Kenya (**Figure 1**). We used a purposive sampling that aligns with the WHO sampling guideline for clinical characterization of COVID-19 pandemic within a country to select the isolation sites [12] and considered all the eight administrative provinces during the selection of the isolation centres to ensure a national representation. We selected counties with high caseloads in each province (taking into consideration POEs) and included the main isolation centres for every selected county. All available case files at the selected isolation centres were reviewed.

A COVID-19 case was defined as any patient admitted into an isolation centre with a positive reverse transcriptase polymerase chain reaction (RT-PCR) test result for SARS-CoV-2 in Kenya. The cases were categorized as symptomatic or asymptomatic cases based on clinical syndromes at the time of admission. An asymptomatic COVID-19 case was defined any patient admitted at the isolation centre but without clinical symptoms or signs. A symptomatic COVID-19 case was defined as any patient who was admitted at an isolation centre with a record of any of the following clinical presentations on admission: fever, cough, difficulty in breathing, running nose, sore throat, headache, myalgia, anosmia, fatigue, diarrhoea, nausea, vomiting, irritability, confusion, pharyngeal exudate, conjunctival injection, seizures, or altered consciousness. We further classified the symptomatic cases into severe and non-severe cases based on their clinical course. A non-severe case was a COVID-19 symptomatic case that did not require oxygen supplementation while a severe case required oxygen supplementation. The cases were also grouped into survivors (those discharged alive from the isolation centre) and non-survivors (died while in isolation).

### Data collection

Data collectors gathered the patients’ personal information, residence, occupation, comorbidities, symptoms, clinical profiles, likely exposures, and case contacts using an open data kit (ODK) application-based abstraction tool installed on tablets. Each data collector was issued with a tablet with the abstraction tool pre-installed, trained, and pre-tested on the data collection process. During the field activity, each team was assigned a field supervisor to provide support. Personal information abstracted included age, sex, nationality, and completed level of education; residence information abstracted from the patients included country, county, sub-county, ward, and village; occupation variables were health worker, veterinarian, transport, tourism, and hospitality sectors; co-morbidities considered were pregnancy, heart disease, hypertension, diabetes, liver disease, chronic lung disease, neurological disease, immunodeficiency conditions, renal disease, and drug use. The symptoms variables included symptoms on admission, date of onset, fever, general malaise, cough, sore throat, running nose, difficulty in breathing, diarrhoea, nausea, vomiting, loss of appetite, loss of smell (anosmia), loss of taste (ageusia), and headache. The clinical profiles were fever (°C) on admission, highest fever (°C) recorded, respiratory rate (/min), oxygen saturation (SpO2), upper respiratory tract infections (URTI), pneumonia, acute respiratory distress syndrome (ARDS), sepsis, cardiac disease, thromboembolic events, neurological conditions, ocular manifestations, renal disease, and survivorship. The likely exposures 14 days prior to illness included were international travel, local travel, health care setting, contact with a case, and gathering.

### Data analysis

The data were downloaded and cleaned in Microsoft Excel spreadsheets and analysed using both Epi Info 7 (US CDC, 2020) and Stata statistical software 17 (StataCorp LLC. 2021). Socio-demographic data (personal information, residence, and occupation), co-morbidities, symptoms, clinical profile, exposures, and contacts were summarized using median and mean for continuous variables and proportions for categorical variables. The socio-demographics, co-morbidities, symptoms, exposures, and contacts were assigned exposure variables, while the clinical spectrums (symptoms and clinical profiles) assigned outcome variables, and bivariate analysis done to assess their associations. The exposure variables with *P* ≤ 0.20 from the bivariate analysis were included in an unconditional logistic regression model using the backward elimination method at a *P* = 0.05 level of significance [13]. The model was run and variables with *P* > 0.05 level of confidence were dropped. The process was repeated until only variables with *P* ≤ 0.05 remained in the final model. The variables retained in the model were considered independently associated with the outcome variables.

## RESULTS

**Figure 2:**
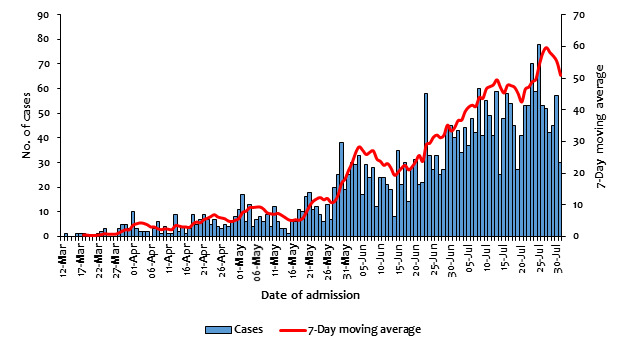
Distribution of cases of COVID-19 by date of admission during the early phases of the pandemic in Kenya.

A total of 2796 case records were reviewed from health facility-based isolation centres (n = 2359 (84.4%)), standalone institutional isolation units (n = 389 (13.9%)), and home-based isolation and care (HBIC) (n = 48 (1.7%)). The cases included 2049 (73.3%) males, 852 (30.5%) persons aged 30-39 years, 2730 (97.6%) Kenyans, 532 (19.0%) with tertiary level education, 636 (34.8%) transport sector employees, and 743 (26.6%) Nairobi City County residents (**Table 1**). The data demonstrated an outbreak that began on the March 13, 2020, and steadily increased with a small spike in late May 2020 to early June 2020 and finally peaked in the last week of July 2020 with a daily average caseload of 60 cases (**Figure 2**).

**Table 1:** Distribution of COVID-19 cases by sex, age, nationality, occupation, and residence during the early phase of the pandemic in Kenya, 2021

Variable	Frequency (n)	Proportion (%)
**Sex**		
Male	2049	73.3
Female	747	26.7
**Age group (years)**		
0-9	69	2.5
10-19	77	2.8
20-29	603	21.6
30-39	852	30.5
40-49	578	20.7
50-59	362	12.9
>60	255	9.1
**Nationality**		
Kenyan	2730	97.6
Non-Kenyans	66	2.4
**Race**		
Blacks	2748	98.3
Asian	42	1.5
Caucasian	6	0.2
**Level of education**		
None	508	18.2
Informal	59	2.1
Primary	144	5.2
Secondary	215	7.7
Tertiary	532	19.0
Not Indicated	1338	47.9
**Occupation**		
Transport	636	34.8
Business	194	10.6
Health worker	174	9.5
Minor	135	7.4
Hospitality	107	5.9
Semi-skilled labour	81	4.4
Judiciary	76	4.2
Technician	72	3.9
Disciplined forces	64	3.5
Farmer	52	2.8
Finance officer	47	2.6
Education	42	2.3
House help	41	2.2
Civil servant	34	1.9
Manager	25	1.4
Elderly	23	1.3
Humanitarian	18	1.0
Religious leader	6	0.3
**County of usual residence**		
Nairobi	743	26.6
Mombasa	311	11.1
Kajiado	259	9.3
Kiambu	228	8.2
Migori	228	8.2
Machakos	177	6.3
Uasin Gishu	105	3.8
Others*	745	26.6
**Total**	2796	100.0

*Includes Nakuru, Nyeri, Garissa, Busia, Laikipia, Muranga, Wajir, Kisumu, Meru, Kitui, Lamu, Makueni, Mandera, Kakamega, Kilifi, Kwale, Taita Taveta, Tana River, Siaya, Nyandarua, Kericho, Kisii, Trans Nzoia, Homa Bay, Nandi, Marsabit, Vihiga, Bungoma, Isiolo, Baringo, Embu, Narok, Bomet, Kirinyaga, Nyamira, Elgeyo Marakwet, and Tharaka-Nithi.

Up to 1200 (42.9%) cases had symptoms, including 443 (36.9%) cases on admission and 757 (63.1%) cases while at the isolation centres (**Table 2**). Among the 757 cases who developed symptoms while at the isolation centres, 258 (34.1%) had severe disease, while 50 (6.6%) did not survive it. The symptoms included cough (n = 572 (47.7%)), difficulty in breathing (n = 383 (31.9%)), fever (n = 332 (27.7%)), among others. Six hundred and nine (21.8%) cases had at least one underlying medical condition, out of which 129 (21.2%) had more than one (>1). The underlying medical conditions included hypertension (n = 260 (42.7%)) and diabetes (n = 211 (34.6%)), among others. Up to 1893 (67.7%) cases had reports on the possible sources of exposure; 1013 (53.5%) through travel, 498 (26.3%) through contact with a case, and 181 (9.6%) during community gatherings, among others. From the reviewed records, 2340 (83.7%) cases had their contacts traced, 2219 (98.4%) cases had no contacts, while the 121 (5.2%) cases with contacts had a median of three contacts (range = 1-66). A total of 760 contacts were listed, 632 (83.2%) of which were tested, with 118 (18.7%) testing positive. Among the 1200 symptomatic cases, 372 (31.0%) had severe outcomes. At the time of the records review, 1946 (69.6%) cases had been discharged from the isolation centres out of which 1866 (66.7%) were discharged alive (recoveries) while 80 cases had died (CFR = 2.8%).

**Table 2:** Distribution of COVID-19 cases by symptoms, underlying conditions, possible exposures, contacts, and outcomes during the early phase of the pandemic in Kenya, 2021

Variables	Frequency	Proportion (%)
**Symptomatology (n = 1200)**		
Symptomatic on isolation	443	36.9
Developed symptoms in isolations	757	63.1
Reported symptoms:		
Cough	572	47.7
Difficulty in breathing	383	31.9
Fever (≥38.0°C)	332	27.7
General malaise	178	14.8
Headache	171	14.3
Sore throat	116	9.7
Loss of appetite	56	4.7
Running nose	48	4.0
Vomiting	47	3.9
Diarrhoea	39	3.3
Nausea	30	2.5
Anosmia	27	2.3
Ageusia	21	1.8
Chest pain	11	0.9
Epigastric pain	6	0.5
Urine retention	2	0.2
Convulsions	1	0.1
**Underlying conditions (n = 609)**		
At least 1 underlying medical condition	609	21.8
>1 underlying medical condition	129	4.6
Type of underlying medical condition:		
Hypertension	260	42.7
Diabetes	211	34.6
HIV	86	14.1
Chronic lung disease	56	9.2
Pregnancy	31	5.1
Drug/substance abuse	30	4.9
Neurological disease	28	4.6
Heart disease	25	4.1
Renal disease	22	3.6
Liver disease	13	2.1
**Possible sources of exposure (n = 2769)**		
Indicated	1893	67.7
Not indicated	903	32.3
Possible sources of exposure:		
Travel	1013	53.5
Contact with a case	498	26.3
Community gatherings	181	9.6
Healthcare setting	118	6.2
Workplace	49	2.6
Prison	34	1.8
**Contacts (n = 2769)**		
Cases with contacts traced:	2340	83.7
Cases with contacts identified	121	5.2
Cases with no contacts identified	2219	94.2
Cases with no contacts traced	456	16.3
**Outcomes**		
Symptomatology (n = 2796):		
Symptomatic	1200	42.9
Asymptomatic	1596	57.1
Severity (n = 1200):		
Severe cases	372	31.0
Non-severe cases	828	69.0
Survivorship (n = 2796):		
Cases discharged from isolation units	1946	69.6
Cases discharged alive (survivor)	1866	95.9
Cases discharged dead (non-survivors)	80	4.1
Cases still admitted in isolation units	850	30.4

The odds of developing COVID-19 symptoms was higher among case aged above 60 years (odds ratio (OR) = 1.99, *P* = 0.007), males (OR = 1.47, *P* = 0.019), cases with at least one (OR = 2.73, *P* < 0.001) or more than one underlying medical condition (OR = 1.76, *P* = 0.023), or cases who had been exposed to the disease through the community gatherings (OR = 1.93, *P* = 0.003) compared to those who were not (**Table 3**). The odds of developing COVID-19 symptoms were lower among cases who worked in the disciplined forces (OR = 0.47, *P* = 0.009), transport sector employees (OR = 0.31, *P* = 0.000), drugs and substance users (OR = 0.11, *P* < 0.001), those with neurological disorders (OR = 0.20, *P* < 0.001), and those with heart diseases (OR = 0.16, *P* < 0.001) compared to those who were not. The COVID-19 cases who had at least one underlying medical condition (OR = 1.56, *P* < 0.001) or more than one underlying medical condition (OR = 1.66, *P* = 0.030) had higher odds of developing severe COVID-19 disease compared to those who were not (**Table 4**). The odds of developing severe COVID-19 disease were lower among cases who were male (OR = 0.51, *P* < 0.001), exposed through community gatherings (OR = 0.27, *P* < 0.001), or exposed as contacts of confirmed cases (OR = 0.41, *P* < 0.001) compared to those who were not. The odds of survival of cases from COVID-19 disease was higher among transport sector employees (OR = 3.35, *P* = 0.004) and those who were exposed through contacts with confirmed cases (OR = 3.98, *P* = 0.009) compared to those who are not (**Table 5**). However, cases who were aged ≥60 years (OR = 0.58, *P* = 0.034), had at least one underlying medical condition (OR = 0.58, *P* = 0.025), were pregnant (OR = 0.18, *P* = 0.016), had liver disease (OR = 0.27, *P* = 0.001), were exposed through travelling (OR = 0.18, *P* < 0.001), were symptomatic (OR = 0.05, *P* < 0.001), and had severe COVID-19 disease (OR = 0.01, *P* < 0.001) were at lower odds of survival compared to those who were not.

**Table 3:** Factors associated with the development of COVID-19 symptoms during early phases of the pandemic in Kenya

Variables	Yes			No			Bivariate analysis			Multivariate analysis		
**Symptomatic**	**Asymptomatic**	**Odds**	**Symptomatic**	**Asymptomatic**	**Odds**	**OR**	**95% CI**	***P*-value**	**OR**	**95% CI**	***P*-value**
**Socio-demographic**												
Aged ≥60 y	179	76	2.36	1021	1520	0.67	3.51	2.65-4.64	<0.001	1.99	1.21-3.29	0.007
Male sex	348	399	0.87	852	1197	0.71	1.23	1.04-1.45	0.018	1.47	1.06-2.03	0.019
Black race	1166	1582	0.74	34	14	2.43	0.30	0.16-0.57	<0.001	0.56	0.21-1.50	0.252
Kenyan national	1559	1171	1.33	37	29	1.28	1.04	0.64-1.71	0.865	-	-	-
**Occupation**												
Disciplined forces	38	94	0.40	656	1039	0.63	0.64	0.43-0.94	0.024	0.47	0.26-0.82	0.009
Health worker	91	91	1.00	603	1042	0.58	1.73	1.27-2.35	<0.001	1.29	0.83-1.93	0.265
Hospitality	45	62	0.73	649	1071	0.61	1.20	0.81-1.78	0.371	-	-	-
Public service	83	103	0.81	611	1030	0.59	1.36	1.00-1.84	0.049	1.07	0.70-1.63	0.769
Transport	139	497	0.28	555	636	0.87	0.32	0.26-0.40	<0.001	0.31	0.23-0.42	<0.001
**Underlying medical conditions**												
Having at condition	400	209	1.91	800	1387	0.58	3.32	2.75-4.01	<0.001	2.73	2.01-3.71	<0.001
Multiple (>1) condition	92	37	2.49	308	172	1.79	1.39	0.91-2.12	0.129	1.76	1.08-2.87	0.023
Type of medical condition												
*Hypertension*	178	82	2.17	222	127	1.75	1.24	0.88-1.75	0.212	-	-	-
*Diabetes mellitus*	154	57	2.70	246	152	1.62	1.67	1.16-2.40	0.006	1.17	0.77-1.79	0.463
*Immunosuppression*	61	25	2.44	339	184	1.84	1.32	0.80-2.18	0.269	-	-	-
*Chronic lung disease*	38	18	2.11	362	191	1.90	1.11	0.62-2.00	0.719	-	-	-
*Pregnancy*	24	6	4.00	376	203	1.85	2.16	0.84-6.56	0.114	1.73	0.68-4.40	0.250
*Drug abuse*	6	24	0.25	394	185	2.13	0.18	0.04-0.30	<0.001	0.11	0.04-0.27	<0.001
*Neurological disorders*	9	19	0.47	391	190	2.06	0.23	0.10-0.52	<0.001	0.20	0.09-0.45	<0.001
*Heart disease*	9	16	0.56	391	193	2.03	0.28	0.12-0.64	0.001	0.16	0.07-0.40	<0.001
*Renal disease*	15	7	2.14	385	202	1.91	1.12	0.42-3.31	1.000	-	-	-
*Liver disease*	11	2	5.50	389	207	1.88	2.93	0.63-27.38	0.236	-	-	-
**Exposures**												
Travel	373	646	0.58	388	485	0.80	0.72	0.60-0.87	<0.001	0.81	0.62-1.06	0.131
Community	103	77	1.34	658	1054	0.62	2.14	1.57-2.92	<0.001	1.93	1.26-2.95	0.003
Contact	211	288	0.73	550	844	0.65	1.12	0.91-1.38	0.269	-	-	-

OR – odds ratio, CI – confidence interval

**Table 4:** Factors associated with severity of COVID-19 during early phases of the pandemic in Kenya, May-July 2020

Variables	Yes			No			Bivariate analysis			Multivariate analysis		
**Severe**	**Non-severe**	**Odds**	**Severe**	**Non-severe**	**Odds**	**OR**	**95% CI**	***P*-value**	**OR**	**95% CI**	***P*-value**
**Socio-demographic**												
Aged ≥60 y	44	134	0.33	328	693	0.47	0.69	0.48-0.99	0.044	0.53	0.26-1.10	0.086
Black race	362	804	0.45	10	24	0.42	1.08	0.51-2.28	0.839	-	-	-
Kenyan national	362	809	0.45	10	19	0.53	0.85	0.39-1.85	0.681	-	-	-
**Occupation**												
Disciplined forces	16	22	0.73	199	457	0.44	1.67	0.86-3.25	0.127	0.65	0.30-1.42	0.281
Health worker	25	66	0.38	190	413	0.46	0.82	0.50-1.35	0.438	-	-	-
Hospitality	15	30	0.50	200	449	0.45	1.12	0.59-2.13	0.724	-	-	-
Public service	19	64	0.30	196	415	0.47	0.63	0.37-1.08	0.089	0.80	0.28-2.25	0.687
Transport	41	98	0.42	174	381	0.46	0.92	0.61-1.38	0.672	-	-	-
**Underlying medical conditions**												
Having at condition	148	252	0.59	224	576	0.39	1.51	1.17-1.95	0.001	1.56	1.20-2.01	<0.001
Multiple (>1) condition	44	48	0.92	104	204	0.51	1.80	1.12-2.88	0.014	1.66	1.05-2.64	0.030
Type of medical condition												
*Hypertension*	70	108	0.65	78	144	0.54	1.20	0.80-1.80	0.388	-	-	-
*Diabetes mellitus*	54	100	0.54	94	152	0.62	0.87	0.57-1.33	0.526	-	-	-
*Immunosuppression*	27	34	0.79	121	218	0.56	1.43	0.82-2.48	0.202	-	-	-
*Chronic lung disease*	13	25	0.52	135	227	0.59	0.87	0.43-1.77	0.708	-	-	-
*Pregnancy*	14	10	1.40	134	242	0.55	2.53	1.10-5.85	0.026	1.40	0.62-3.15	0.416
*Drug abuse*	3	3	1.00	145	249	0.58	1.72	0.23-12.97	0.674	-	-	-
*Neurological disorders*	2	7	0.29	146	245	0.60	0.48	0.05-2.57	0.495	-	-	-
*Heart disease*	2	7	0.29	146	245	0.60	0.48	0.05-2.57	0.495	-	-	-
*Renal disease*	7	8	0.88	141	244	0.58	1.51	0.46-4.89	0.428	-	-	-
*Liver disease*	5	6	0.83	143	246	0.58	1.43	0.33-5.74	0.544	-	-	-
**Exposures**												
Travel	118	255	0.46	67	321	0.21	2.22	1.58-3.12	<0.001	0.79	0.58-1.06	0.115
Community	13	90	0.14	172	486	0.35	0.41	0.22-0.75	0.003	0.27	0.15-0.49	<0.001
Contact	37	174	0.21	148	402	0.37	0.58	0.39-0.86	0.007	0.41	0.28-0.60	<0.001

OR – odds ratio, CI – confidence interval

**Table 5:** Factors associated with survivorship from COVID-19 during early phases of the pandemic in Kenya

Variables	Yes			No			Bivariate analysis			Multivariate analysis		
**Survivor**	**Non-survivor**	**Odds**	**Survivor**	**Non-survivor**	**Odds**	**OR**	**95% CI**	***P*-value**	**OR**	**95% CI**	***P*-value**
**Socio-demographic**												
Aged ≥60 y	190	17	11.18	1676	63	26.60	0.42	0.24-0.73	0.002	0.58	0.30-0.95	0.034
Black race	1827	78	23.42	39	2	19.50	1.20	0.14-4.79	0.684	-	-	-
Kenyan national	1824	78	23.38	42	2	21.00	1.11	0.13-4.42	0.702	-	-	-
**Occupation**												
Disciplined forces	87	4	21.75	1143	33	34.64	0.63	0.22-2.50	0.333	-	-	-
Health worker	125	4	31.25	1105	33	33.48	0.93	0.32-3.69	0.785	-	-	-
Hospitality	73	3	24.33	1157	34	34.03	0.72	0.22-3.73	0.483	-	-	-
Public service	128	3	42.67	1102	34	32.41	1.32	0.40-6.79	1.000	-	-	-
Transport	410	7	58.57	820	30	27.33	2.14	0.93-4.92	0.066	3.35	1.47-7.62	0.004
**Underlying medical conditions**												
Having at condition	436	27	16.15	1430	53	26.98	0.60	0.37-0.96	0.033	0.58	0.36-0.93	0.025
Multiple (>1) condition	93	5	18.60	343	22	15.59	1.19	0.43-4.14	1.000	-	-	-
Type of medical condition												
*Hypertension*	193	8	24.13	243	19	12.79	1.89	0.81-4.40	0.136	1.90	0.81-4.44	0.138
*Diabetes mellitus*	162	9	18.00	274	18	15.22	1.18	0.52-2.69	0.690	-	-	-
*Immunosuppression*	62	5	12.40	374	22	17.00	0.73	0.26-2.56	0.571	-	-	-
*Chronic lung disease*	42	2	21.00	394	25	15.76	1.33	0.31-12.00	1.000	-	-	-
*Pregnancy*	21	3	7.00	415	24	17.29	0.40	0.11-2.27	0.157	0.18	0.04-0.75	0.016
*Drug abuse*	5	1	5.00	431	26	16.58	0.30	0.03-14.81	0.305	-	-	-
*Neurological disorders*	20	1	20.00	416	26	16.00	1.25	0.81-53-78	1.000	-	-	-
*Heart disease*	16	2	8.00	420	25	16.80	0.48	0.10-4.51	0.283	-	-	-
*Renal disease*	14	0	0.00	422	27	15.63	-	-	-	-	-	-
*Liver disease*	9	2	4.50	427	25	17.08	0.26	0.05-2.65	0.130	0.27	0.06-1.33	0.001
**Exposures**												
Travel	732	40	18.30	623	8	77.88	0.23	0.11-0.51	0.000	0.18	0.08-0.39	<0.001
Community	153	4	38.25	1202	44	27.32	1.40	0.50-5.44	0.278	0	0	0
Contact	348	4	87.00	1007	44	22.89	3.80	1.37-14.66	0.006	3.98	1.41-11.25	0.009
**Clinical profiles**												
Symptomatic	896	77	11.64	970	3	323.33	0.04	0.01-0.11	<0.001	0.05	0.02-0.17	<0.001
Severe case	192	76	2.53	704	1	704.00	0.01	0.00-0.02	<0.001	0.01	0.00-0.04	<0.001

OR – odds ratio, CI – confidence interval

## DISCUSSION

The early phase of COVID-19 pandemic in Kenya demonstrated heavy caseloads among the male, middle aged, transporters, and residents of Nairobi and Mombasa counties. The transmission of SARS-CoV-2 during the early phase was driven by travellers and sporadic isolated cases at the main POE in Nairobi and Mombasa counties. Men had a higher risk of contracting the infection, which was not surprising, as they constitute most of the travellers and spend most of the time outdoors due to social or livelihood activities. They also have a higher prevalence of smoking and alcohol consumption[14-16]. Kenya’s strategy of testing international travellers at the POEs, implementing movement restrictions from Nairobi and Mombasa Counties (which were considered hotspots), targeting the hotspots for mass testing, and suspending international passenger flights could explain the larger caseloads being Kenyans, travellers, and residents of the major POE [4,17]. The trend of cases in the study coincided with the national picture of COVID-19 cases, reaffirming the representativeness of the study group [4].

The profile of symptoms noted during this study cut across several systems, including respiratory, cardiovascular, gastrointestinal, hepatobiliary, musculoskeletal, neurological, urinary, and reproductive systems similar to observations in other settings [18-20]. This picture of symptoms involving multiple systems renders the basic syndromic screening less specific [21,22]. Few cases in this study were symptomatic on admission, consistent with findings from other countries [23,24]. However, many asymptomatic cases developed symptoms while in isolation and progressed to severe states of the disease or death. This phenomenon was documented in other settings and necessitates close monitoring of patients on HBIC [25,26]. The high yield of asymptomatic cases of COVID-19 is indicative of effective surveillance, screening, and testing strategies, which is important considering that asymptomatic cases have been linked to resurgence [26,27]. This poses a big challenge to the low to middle income countries, which may not be able to implement robust screening strategies.

This study also described chronic medical conditions and infections which, if comorbid with COVID-19, could result in unfavourable outcomes, consistent with studies within and outside the African settings [28,29]. This requires that those living with these conditions obtain early screening, close monitoring, and prompt management to minimize chances of unfavourable outcomes, should they contract the disease. During the early phase of the pandemic in Kenya, transmission was largely driven by importation of cases. Contact tracing is a key measure for containing infectious diseases such as COVID-19 [30]. We observed good contact tracing during the early phases, but with very poor yields, considering that travellers, transporters, and community gatherings make many contacts. This poor yield could be attributed to the public health and social measures, such as evacuation of contacts using ambulances with sirens and backed up by security personnel, mandatory quarantine at designated sites at the cost of contacts, and associated stigma which were deterrent to disclosure of contacts [4,31,32]. Due to the high positivity rates observed among the contacts, the poor yields occasioned by the deterrence could have worsened the transmission within the populations.

The presence of disease symptoms plays a fundamental surveillance role in identifying illness or clusters early before confirmation, which supports rapid response, thereby reducing associated morbidity and mortality [33,34]. Older adults were more likely to develop clinical manifestations of COVID-19 compared to younger age groups due to their immune response possibly being blunted and underlying chronic illnesses possibly also augmenting the signs of infection [35]. Male cases were shown to be more likely to become symptomatic than female cases. This has been attributed to several possible factors, such as the higher expression of angiotensin-converting enzyme-2 (the receptor for SARS-CoV-2) in males than females, the sex-based immunological differences driven by sex hormones and the X-chromosome [36,37], and possibly even lifestyle differences (higher levels of smoking, drinking, and drug abuse among men compared to women) [36,38]. This study found that many asymptomatic COVID-19 cases upon diagnosis could be attributed to the testing strategy targeting high risk groups such as travellers, transporters, close contacts, etc. This testing strategy should be effective in stopping transmission if coupled with mandatory isolation, since both the asymptomatic and pre-symptomatic are capable of transmitting the disease [39,40]. Due to the high number of cases converting from asymptomatic to symptomatic, severe, and even dying within the isolation centres, cases on HBIC should be monitored closely to minimize the occurrence of adverse outcomes.

Having at least one underlying medical condition was associated with severe disease outcomes. These findings align with other studies that have demonstrated that cases with comorbidities are at a higher risk of severe COVID-19 outcomes (including death), as they impair the body’s ability to fight off disease [41,42]. The higher the number of comorbidities, the worse the outcomes [43]. The pandemic happening in settings with a high prevalence of chronic diseases can overwhelm health care systems. The strategy of mandatory testing and isolation of positive cases within health care settings meant early diagnosis and close monitoring reducing the risk of severe COVID-19 disease among the travellers, transporters, and contacts as well.

The high CFR among cases in isolation centres when compared to the general population may be attributed to the mandatory isolation and its attendant psychological, social, and economic distress (which worsens health outcomes) [4,44,45]. Work stress among the health care workers and pressure on limited essential supplies needed to manage cases could also have contributed to this high CFR [46,47]. Advanced age, underlying medical conditions (including pregnancy), and severe cases were less likely to survive the disease consistent with previous studies [48,49]. Similarly, early recognition (as in the case of mandatory screening of risk groups such as travellers or contacts)and prompt intervention have been shown to reduce mortality associated with COVID-19 [50].

### Limitations

This study was based on record reviews from different COVID-19 isolation centres, so there may be variations in the way data was gathered and recorded, limiting the extraction and interpretation of the variables. Some records were incomplete or lost in the course of time, leading to missing data.

## CONCLUSIONS

This characterization of COVID-19 cases in Kenya in the early phase of the pandemic observed that mandatory testing of risk groups and isolation of positive cases was an effective control strategy, as many cases were potential asymptomatic spreaders. Public health and social measures in response to the COVID-19 pandemic need to be carefully implemented, without jeopardizing other critical response functions such as contact tracing. Many cases initially classified as asymptomatic later developed symptoms with severe and non-survivor outcomes which has implications on the selection and close monitoring of cases put under HBIC. The elderly and those with underlying medical conditions (comorbidities) are at higher risk of COVID-19 morbidities and mortalities, hence there is a need to target these groups with prevention and control measures.
